# Classification bias in commercial business lists for retail food stores in the U.S.

**DOI:** 10.1186/1479-5868-9-46

**Published:** 2012-04-18

**Authors:** Euna Han, Lisa M Powell, Shannon N Zenk, Leah Rimkus, Punam Ohri-Vachaspati, Frank J Chaloupka

**Affiliations:** 1 College of Pharmacy, Gachon University, 534-2 Yeonsu3-dong, Yeonsu-gu, Incheon, 406-799, Korea; 2 Institute for Health Research and Policy & Department of Economics, University of Illinois at Chicago, 1747 West Roosevelt Road, Chicago, IL, 60608, USA; 3 College of Nursing, University of Illinois at Chicago, 845 South Damen Avenue MC 802, Chicago, IL, 60612, USA; 4 School of Nutrition and Health Promotion, Arizona State University, 500 N. Third Street, Phoenix, AZ, 85004, USA; 5 Department of Economics & Institute for Health Research and Policy, University of Illinois at Chicago, 1747 West Roosevelt Road, Chicago, IL, 60608, USA

**Keywords:** Commercial business lists, Food stores, Classification error

## Abstract

**Background:**

Aspects of the food environment such as the availability of different types of food stores have recently emerged as key modifiable factors that may contribute to the increased prevalence of obesity. Given that many of these studies have derived their results based on secondary datasets and the relationship of food stores with individual weight outcomes has been reported to vary by store type, it is important to understand the extent to which often-used secondary data correctly classify food stores. We evaluated the classification bias of food stores in Dun & Bradstreet (D&B) and InfoUSA commercial business lists.

**Methods:**

We performed a full census in 274 randomly selected census tracts in the Chicago metropolitan area and collected detailed store attributes inside stores for classification. Store attributes were compared by classification match status and store type. Systematic classification bias by census tract characteristics was assessed in multivariate regression.

**Results:**

D&B had a higher classification match rate than InfoUSA for supermarkets and grocery stores, while InfoUSA was higher for convenience stores. Both lists were more likely to correctly classify large supermarkets, grocery stores, and convenience stores with more cash registers and different types of service counters (supermarkets and grocery stores only). The likelihood of a correct classification match for supermarkets and grocery stores did not vary systemically by tract characteristics whereas convenience stores were more likely to be misclassified in predominately Black tracts.

**Conclusion:**

Researches can rely on classification of food stores in commercial datasets for supermarkets and grocery stores whereas classifications for convenience and specialty food stores are subject to some systematic bias by neighborhood racial/ethnic composition.

## Background

As the prevalence of obesity has grown tremendously in the United States over the past few decades [1,2], numerous studies have been conducted to understand the obesity epidemic. Aspects of the food environment such as the availability of different types of food stores have recently emerged as key modifiable factors that may contribute to the increased prevalence of obesity, particularly in low-income neighborhoods [3-5]. Previous studies have examined associations of food store densities with neighborhood socioeconomic, racial, and ethnic characteristics and with body weight outcomes. Research has found that low-income neighborhoods and predominately African American and Latino neighborhoods have fewer supermarkets and more convenience stores than higher income and predominately White neighborhoods [6]. Although results are mixed, several studies have reported that the availability of supermarkets was associated with lower body mass index (BMI) for children and adolescents [7-10] as well as adults [11-13], particularly those of low-socioeconomic status. At the same time, studies have found that greater availability of convenience stores is associated with higher BMI, particularly among low-income adult women [10,12].

Many of these studies have derived their results based on secondary datasets including proprietary commercial business lists, telephone directories, public administrative data from a local health department, or census data. Measuring the food environment based on secondary data sources is often inevitable due to study design and resource constraints especially in large scale research studies. Yet, these secondary data listings are primarily created for business purposes and may not require the same level of precision in classification as needed for research. The relationship between the food environment and individual food consumption or body weight outcomes is reported to vary by store type. However, the validity of these relationships relies on the assumption of no systematic misclassification of store type in the databases used in those studies. Therefore, it is important to understand the extent to which such secondary data sources correctly classify food stores particularly given some of the mixed results observed in the relationships between store types and body weight outcomes. Only a few studies have validated secondary data sources [13-17] and to our knowledge only one study [18] conditioned their validation of count error on correct classification of outlet type.

Our study builds on this previous literature by investigating the extent of classification error for food stores in two secondary commercial data sources that are widely available in the U.S. We compared Dun and Bradstreet (D&B) and InfoUSA to ground-truthed data in the Chicago Metropolitan Statistical Area (MSA) (referred to hereafter as the Chicago MSA). Detailed store attributes were collected inside each retail outlet to accurately identify store type. We analyzed whether outlet attributes differed between food stores that were correctly classified and those misclassified by store type to identify any specific patterns of mis-classification in the two business lists. Finally, we assessed systematic biases in the accuracy of store classification by neighborhood characteristics in multivariate analyses.

## Methods

### Data and measures

Our sample included 278 urban census tracts (used as a proxy for neighborhoods) that were randomly drawn from the Chicago MSA. Four census tracts were excluded because they contained no businesses on the ground or in the business lists. The final analysis was based on 274 census tracts across 9 counties, covering approximately 5,049 road miles. To ensure diversity with respect to census tract socioeconomic status and racial/ethnic composition, we used a stratified sampling approach. Using 2000 Census data, we measured socioeconomic status using median household income and categorized each tract as low-, middle-, and high-income based on income tertiles. Among the low-income urban tracts, we further stratified tracts by race [predominantly (70%) white, predominantly black and mixed (not predominantly white or black)] and also by ethnicity [predominantly Hispanic and non-Hispanic]. Stratification by race and ethnicity was not done for all income levels because of low cell counts for predominantly minority race and ethnicity in higher income tracts. As a result, we oversampled tracts in the bottom income tertile to ensure adequate sample size for predominately racial/ethnic minority census tracts that are disproportionately low-income, with the final sample including 65%, 18%, and 17% of low, middle, and high income tracts, respectively. In our sample, 40% of low-income tracts were predominately White, 23% predominately Black, and 37% racially/ethnically mixed. Predominately Hispanic tracts comprised 17% of the sample.

The ground survey was undertaken from May through July 2009. Two trained field staff members surveyed the entire census tract to identify any food stores and recorded detailed attributes of those retailers based on direct observations inside the outlets. Field teams were instructed to observe both sides of all streets falling within each tract, but to observe only the interior side of the tracts’ boundary streets. Establishments that had signs but were determined to be permanently closed were not considered valid outlets present on the ground. Further details about the ground truthing are discussed elsewhere [18].

Among all food stores found on the ground, in this analysis we included only those that were also present in the business lists, D&B and InfoUSA, in order to compare classification of those outlets in the business lists to the classification based on direct observation. A total of 612 and 729 food stores identified on the ground were included in D&B and InfoUSA lists, respectively among a total of 1,241 food stores identified on the ground. Food stores found on the ground were classified based on directly observed attributes collected from the ground-survey using the definitions derived from the literature and presented in Table 1.

**Table 1:** Classification of food stores

**Food store classification**	**Ground-survey**	**D&B list**	**InfoUSA list**
	**Store characteristics**	**Primary SIC code**	**Primary SIC code†**
Convenience store	· At most 2 cash register; and, · No fresh meats; and, · Less than 10 fruits and vegetables; and, · Not a specialty food store	· 541102 (Convenience stores) · 55410000 (Gasoline service stations) · 55419901 (Filling stations, gasoline) · 55419903 (Truck Stops)	· 541103 (Convenience stores) · 554101 (Service stations-gasoline & oil) · 554102 (Gas-diesel) · 554103 (Truck stops & plazas)
Supermarket	· 4 or more cash registers; and, · Have at least two full services among butcher, deli, and bakery; and, · 20 or more fruits and vegetables, and; · Have fresh meats, and; · Have fresh milk; and, · Have a fresh produce section; and, · Not a specialty food store	· 541101 (Supermarkets)	· 541101 (Food markets) · 541102 (Snack products) · 541104 (Food products-retail) · 541105 (Grocers-retail) · 541106 (Markets-kosher) · 541107 (Grocers-ethnic foods) · 541108 (Grocers-health foods) · 541108 (Grocers-take-out foods)
Grocery store	· Not a convenience store; and, · Not a supermarket; and, · Not a specialty food store	· 541100 (Grocery stores) · 541199 (Grocery stores, nec)	· Same as supermarket
Specialty food store	· Bakery · Meat/fish stores · Fruit/vegetable stores · Candy/nut stores · Coffee/tea stores · Other specialty stores	· 5421 (Meat and fish markets) · 5431 (Fruit and vegetable market) · 5441 (Candy, nut, and confectionary stores) · 5451 (Dairy products stores) · 5461 (Retail bakeries) · 5499 (Miscellaneous food stores)	· 5421 (Meat and fish markets) · 5431 (Fruit and vegetable market) · 5441 (Candy, nut, and confectionary stores) · 5451 (Dairy products stores) · 5461 (Retail bakeries) · 5499 (Miscellaneous food stores)

† Supermarkets and grocery stores cannot be classified separately in the InfoUSA.

Food stores found on the ground were categorized as supermarket, grocery, convenience, and other specialty stores. We first identified specialty food stores as bakeries, meat or fish stores, fruit or vegetable stores, candy or nut stores, and coffee or tea stores [19]. For the remaining stores, we classified a food store as a supermarket if it had 4 or more cash registers [20]; had two or more independent service departments of butcher, deli, or bakery [21]; sold fresh meat [21,22]; and carried 20 or more fresh fruits and vegetables [23]. Non-specialty food stores with no fresh meat, 10 or fewer fruits and vegetables, and 2 or fewer cash registers were classified as convenience stores [24]. If a food store did not meet the operational definition for a specialty food store, supermarket, or convenience store, we classified it as a grocery store [21,25] (Table 1).

Inter-rater reliability results of store attribute measures, derived from a separate sample of 120 stores, were high. As shown in Table 2, nearly all the items had almost perfect agreement (Kappa score of 0.75 to 1.00). As the lowest score, presence of a butcher or fresh meat counter had lower, but still substantial agreement (Kappa = 0.75). Continuous variables had an Intraclass Correlation Coefficient ranging from 0.92 to 0.97.

**Table 2:** Inter-rater reliability of measures for collecting retailer characteristics

**Measure**	**Statistics**	**Reliability**	**Proportional agreement**
Number of cash registers	Intra Class Correlation	0.87	N/A
Does the store sell/have fresh milk	Simple Kappa	0.94†	0.94
Does the store sell/have fresh meats	Simple Kappa	0.93††	0.96
Does the store have a fresh produce section	Simple Kappa	0.97§	0.99
Number of different types of fresh fruits and vegetables	Intra Class Correlation	0.91‡	N/A
Does the store have a pharmacy	Simple Kappa	1.00	0.97
Does the store have a bank	Simple Kappa	0.92	0.89
Does the storehave a bakery	Simple Kappa	0.95	0.77
Does the storehave a deli	Simple Kappa	0.89	0.96
Does the storehave a butcher or section for unpackaged, fresh meat	Simple Kappa	0.75	0.94

**†**This is the average reliability for the availability of the four different milk types.

**††** This is the average reliability for the availability of regular ground beef and lean ground beef.

**‡** This is the average reliability for the availability of eight different fresh fruit and vegetable items.

**§** This is the average reliability for count of fresh fruits and count of fresh vegetables.

For the secondary data sources, we drew information from two commercial databases, D&B and InfoUSA, which we purchased with a reference date of May 2009. Specifically, we used data on establishments with primary Standard Industrial Classification (SIC) codes falling under SIC 5400 “Grocery Stores”. Primary SIC codes were used as food store classification systems for the D&B and InfoUSA lists. See Table 1 for detailed list of SIC codes used for each business list to define the four store types. All stores meeting these definitions in the lists were geocoded using ArcGIS 9.1 based on their street address.

### Analysis

In order to measure the extent to which classification of retail food stores based on observed characteristics matched the classification denoted by SIC codes in each of the business lists, we calculated the concordance between the on-the-ground and business list food store classification. For each store type, we also performed a two-sided *t*-test to analyze whether store attributes differed among outlets that were correctly classified in the business lists versus outlets that were misclassified in the business lists. Lastly, we explored the extent to which the accuracy in the classification of the store was associated with store attributes and neighborhood characteristics in multivariate regression models. Statistical analyses were performed using STATA 11.0. All the estimates were weighted to account for the sampling design and represent the Chicago MSA.

## Results

### Concordance in classification for food stores

Table 3 shows the concordance in classification for food stores between the on-ground survey and the two business lists. 54% of supermarkets and 64% of grocery stores on the ground were similarly classified in D&B. When we classified supermarkets and grocery stores as one category, 91% of supermarkets and 75% of grocery stores on the ground were listed in that combined category in D&B. In D&B, only 24% of convenience stores on the ground were listed as convenience stores, whereas 45% of them were listed as specialty food stores and 26% were listed as grocery stores. Most specialty food stores (84%) were classified as such in D&B. InfoUSA does not separately classify supermarkets from grocery stores. 81% of supermarkets and 69% of grocery stores on the ground were listed as supermarkets and grocery stores in InfoUSA. In InfoUSA, approximately half of convenience stores on the ground were classified as such, whereas 32% of them were listed as specialty food stores. The classification match rate for specialty food stores in InfoUSA was 85%.

**Table 3:** Concordance in classification between on-ground survey and business lists for food stores

**Classification in business list**	**Classification in ground survey**			
	**Supermarket**	**Grocery store**	**Convenience store**	**Specialty store**
D&B	N = 67	N = 148	N = 293	N = 104
Supermarket	**54%**	11%	5%	1%
Grocery store	**37%**	**64%**	26%	14%
Supermarket & Grocery store	91%	**75%**	31%	15%
Convenience store	0%	6%	**24%**	1%
Specialty food store	9%	19%	45%	**84%**
Total	100%	100%	100%	100%
InfoUSA	N = 73	N = 172	N = 344	N = 140
Supermarket	– †	– †	– †	– †
Grocery store	– †	– †	– †	– †
Supermarket & Grocery store	**81%**	**69%**	19%	14%
Convenience store	1%	13%	**49%**	1%
Specialty food store	18%	18%	32%	**85%**
Total	100%	100%	100%	100%

† Not applicable because InfoUSA does not allow separately identifying supermarket from grocery stores.

‡ Statistics in bold represent for the classification match between ground-survey and the business lists.

### Differences in outlet attributes by classification match

In Table 4, we show the extent to which the observed outlet attributes differed by whether the on-the-ground classification correctly matched the classification in the two commercial lists. Based on the on-ground survey, supermarkets that were correctly classified in D&B had more cash registers and were more likely to have a pharmacy, bank, and bakery. Grocery stores listed as such (versus those that were incorrectly classified) in D&B were more likely to have a fresh produce section, a butcher, and were less likely to have no fresh fruits and vegetables available and to be attached to a gas station. In InfoUSA, the supermarkets/grocery stores that matched with the on-ground survey had more cash registers and were more likely to have fresh meat, a fresh produce section, a pharmacy, a bank, a bakery, a deli, and a butcher. The matched supermarkets/grocery stores in InfoUSA were also less likely to be attached to a gas station and were less likely to sell a limited selection (1–9 different kinds) of fresh fruits and vegetables. Since InfoUSA does not separately classify supermarkets from grocery stores, we also combined the supermarket and grocery store categories in D&B for comparison to InfoUSA. For the combined category of supermarkets and grocery stores, the overall pattern was similar between D&B and InfoUSA. These results imply that both D&B and InfoUSA were more likely to correctly classify larger supermarkets and grocery stores (with more cash registers and various service counters) than smaller supermarkets and grocery stores. Both business lists misclassified some atypical forms of supermarkets or grocery stores such as those attached to a gas station.

**Table 4:** Comparison of food store attributes by match status and food store classification

	**Supermarket**		**Grocery store**		**Supermarket+Grocery store**		**Convenience store**		**Specialty food store**	
	**No match**	**Match**	**No Match**	**Match**	**No Match**	**Match**	**No match**	**Match**	**No match**	**Match**
**D&B**										
Number of cash registers	7.13	10.69***	1.94	1.89	2.70	4.51***	1.31	1.49***	1.18	1.15
Does the store sell/have fresh milk	1.00	1.00	0.96	0.91	0.95	0.95	0.99	1.00	0.24	0.21
Fresh meat	1.00	1.00	0.68	0.81	0.63	0.89***	0.00	0.00	0.53	0.16***
Fresh produce section	1.00	1.00	0.59	0.83***	0.52	0.90***	0.04	0.06	0.24	0.07**
Number of different types of fresh fruits& vegetables : 0	0.00	0.00	0.28	0.07***	0.34	0.04***	0.85	0.50***	0.53	0.92***
Number of different types of fresh fruits& vegetables : 1-9	0.00	0.00	0.15	0.15	0.16	0.09	0.15	0.50***	0.29	0.03***
Number of different types of fresh fruits& vegetables : 10-19	0.00	0.00	0.31	0.35	0.18	0.25	0.00	0.00	0.18	0.01***
Number of different types of fresh fruits& vegetables : ≥20	1.00	1.00	0.26	0.43	0.32	0.63***	0.00	0.00	0.00	0.03
Have a pharmacy	0.03	0.81***	0.00	0.00	0.00	0.18***	0.00	0.00	0.00	0.00
Have a bank	0.06	0.72***	0.00	0.01	0.00	0.17***	0.00	0.00	0.00	0.00
Have a deli	0.58	0.94***	0.04	0.04	0.07	0.32***	0.00	0.00	0.18	0.49**
Have a bakery	1.00	1.00	0.44	0.36	0.48	0.61	0.03	0.03	0.24	0.07**
Have a butcher or unpackaged fresh meat	0.94	0.94	0.58	0.76**	0.53	0.83***	0.00	0.00***	0.41	0.15**
Attached to a gas station	0.00	0.06	0.20	0.00***	0.25	0.01***	0.62	0.30***	0.00	0.00
Attached to a restaurant	0.10	0.11	0.20	0.11	0.18	0.12	0.09	0.04	0.24	0.09
**InfoUSA**										
Number of cash registers	– †		– †		3.09	4.90***	1.12	1.38***	1.24	1.24
Does the store sell/have fresh milk	– †		– †		0.94	0.95	0.99	1.00	0.38	0.17**
Fresh meat	– †		– †		0.72	0.88***	0.00	0.00	0.38	0.09***
Fresh produce section	– †		– †		0.65	0.89***	0.11	0.03***	0.43	0.03***
Number of different types of fresh fruits : 0	– †		– †		0.19	0.05***	0.58	0.81***	0.38	0.97***
Number of different types of fresh fruits : 1-9	– †		– †		0.25	0.07***	0.42	0.19***	0.29	0.01***
Number of different types of fresh fruits : 10-19	– †		– †		0.16	0.24	0.00	0.00	0.19	0.00***
Number of different types of fresh fruits : > = 20	– †		– †		0.40	0.63	0.00	0.00	0.14	0.02
Have a pharmacy	– †		– †		0.07	0.14	0.00	0.00	0.00	0.00
Have a bank	– †		– †		0.06	0.16**	0.00	0.00	0.00	0.00
Have a deli	– †		– †		0.21	0.26	0.00	0.00	0.00	0.53***
Have a bakery	– †		– †		0.51	0.54	0.08	0.02***	0.14	0.05
Have a butcher or unpackaged fresh meat	– †		– †		0.60	0.7**	0.00	0.00	0.00	0.00
Attached to a gas station	– †		– †		0.15	0.02***	0.05	0.67***	0.00	0.00
Attached to a restaurant	– †		– †		0.10	0.11	0.20	0.11	0.09	0.04

** significant at the 5% level, *** significant at the 1% level.

† Not applicable because InfoUSA does not allow separately identifying supermarket from grocery stores.

The patterns for convenience stores were overall not consistent between D&B and InfoUSA. Convenience stores that were correctly classified as such in D&B were less likely to have no fresh fruits and vegetables and be attached to a gas station and more likely to have 1–9 different types of fresh fruits and vegetables. Convenience stores listed as such in InfoUSA were more likely to have no fresh fruits and vegetables and less likely to have a fresh produce section and 1–9 different fresh fruits and vegetables, but more likely to be attached to a gas station (see Table 4). Notably, convenience stores that were correctly classified showed higher number of cash registers for both business lists, which implies that large conveniences stores with more cash registers were likely to be correctly classified. Convenience stores were most frequently misclassified as specialty stores in both business lists.

Matched specialty stores were less likely to have fresh meat, fresh milk (InfoUSA only), a butcher or unpackaged meat (D&B only). Specialty stores found on the ground were also less likely to be classified as such in both business lists in cases where the store had a fresh produce section, 1–9 or 10–19 different types of fresh fruits and vegetables. However, specialty stores with a deli counter were more likely to be listed as specialty stores (Table 5). Results for specialty food stores imply that some atypical forms of specialty food stores carrying fresh fruits and vegetables or fresh meat, such as vegetable markets or meat markets, are likely to be misclassified as either grocery stores or supermarkets.

**Table 5:** Associations of census tract characteristics with the likelihood of classification match by retailer type

	**Food store type**				
	**Supermarket**	**Grocery store**	**Supermarket/Grocery store**	**Convenience store**	**Specialty food store**
**D&B**					
Hispanic	0.4001	1.2639	2.2590**	2.7429***	4.4450***
	(0.2408)	(0.5151)	(0.7506)	(0.9268)	(1.7615)
Black	1.0559	0.7499	0.9300	0.2932***	0.7735
	(0.6008)	(0.2940)	(0.3564)	(0.1030)	(0.2724)
Mixed race	1.4113	0.5684	0.696	0.8327	1.563
	(0.6114)	(0.1852)	(0.2336)	(0.2491)	(0.4864)
Middle income	1.0653	0.966	0.9794	0.841	0.7884
	(0.4812)	(0.2646)	(0.2494)	(0.2053)	(0.2044)
High income	1.2031	1.3043	1.7405	1.0486	0.6093
	(0.6431)	(0.5068)	(0.6929)	(0.3510)	(0.2013)
N	612	612	612	612	612
**InfoUSA**					
Hispanic	– †	– †	1.4512	1.4859	4.9112***
	– †	– †	(0.4282)	(0.4627)	(1.7188)
Black	– †	– †	0.8169	0.5092**	1.6063
	– †	– †	(0.3096)	(0.1632)	(0.5585)
Mixed race	– †	– †	0.8103	1.2273	1.1900
	– †	– †	(0.2522)	(0.3545)	(0.3436)
Middle income	– †	– †	1.5333	1.0041	1.022
	– †	– †	(0.4128)	(0.2352)	(0.2509)
High income	– †	– †	1.4804	1.5749	1.1167
	– †	– †	(0.5057)	(0.4952)	(0.3417)
N	– †	– †	729	729	729

† Not applicable because InfoUSA does not allow separately identifying supermarket from grocery stores.

‡ ** significant at the 5% level, *** significant at the 1% level.

§ White tracts, non-Hispanic tracts, and low income tracts were reference groups.

### Multivariate regression results

Finally, Table 5 presents the multivariate regression results that assessed the extent to which census tract characteristics were associated with the likelihood of a correct classification match by outlet type. The likelihood of a correct classification match for supermarkets and grocery stores did not significantly vary by tract characteristics in either D&B or InfoUSA. One exception was found in the combined category of supermarkets and grocery stores in D&B where we found a positive association of predominately Hispanic tracts with the likelihood of correct classification. However, unless one would need to combine the two categories purposefully, this combined category is only useful in comparing D&B to InfoUSA in terms of the systematic classification bias. The likelihood of a correct classification match for convenience stores was lower by 70% and 49% for D&B and InfoUSA, respectively, in predominately Black tracts compared to White tracts whereas it was 2.7 times higher (D&B only) in Hispanic tracts than non-Hispanic tracts. For specialty food stores, the likelihood of classification match was 4–5 times higher in Hispanic tracts for both business lists compared to non-Hispanic tracts.

## Discussion

The quality of secondary data sources in evaluating the food environment is important in order to reach credible conclusions when using such databases [15]. While business owners are usually required to classify themselves using SIC or The North American Industry Classification System (NAICS) codes (with a possibility of selecting multiple categories) when they register in a commercial database, the validity of their classification is not known [13]. Despite the fact that they have been frequently used to assess the food environment with regard to the obesity epidemic, the validity of commercial business lists has not received adequate attention in the literature. We could locate only a handful of previous studies that validated secondary data for the food environment on the ground [13,15-17,26,27]. No previous studies directly assessed the extent of classification error in commercial business lists. To do so, we used detailed store attributes collected on the ground to determine the type of each food store and delved into the specific component of classification error in two widely used commercial databases in the United States, identifying store and neighborhood characteristics that were associated with classification error.

Assessing whether the classification bias for food stores in secondary data sources is systematic by neighborhood characteristics is important. If, for example, secondary data systematically misclassify convenience stores as grocery stores in Black neighborhoods, and if individuals in Black neighborhoods have higher obesity prevalence than other neighborhoods, researchers may erroneously conclude that no association is found between convenience store availability and weight outcomes when perhaps an inverse relationship exists. Therefore, it is important to assess the extent of systematic bias in the classification error by neighborhood characteristics. Our multivariate regression models in fact showed that the likelihood of correctly classifying supermarkets and grocery stores in either D&B or InfoUSA did not vary by tract characteristics. However, in both business lists, the likelihood of a correct classification match for convenience stores was statistically significantly lower in Black census tracts as compared to White tracts. Correct classification matches for convenience stores (D&B only) and specialty food stores were significantly higher in Hispanic tracts compared to non-Hispanic tracts.

Our results show that the overall validity of food store classification was moderate for both D&B and InfoUSA. Both commercial lists performed moderate to well in correctly classifying supermarkets and grocery stores and correctly classified the majority of specialty food stores. Overall, D&B showed less classification error than InfoUSA for supermarkets and grocery stores, whereas InfoUSA had less classification error for convenience stores. Most importantly, no systematic bias in terms of neighborhood characteristics was found in whether supermarkets and grocery stores were correctly classified for both commercial lists. It should be noted that one important caveat of InfoUSA is that it did not allow users to separately identify supermarkets from grocery stores in their classification system. However, previous studies have reported differences between supermarkets and smaller grocery stores in terms of provision of healthy foods and geographic distributions [24,28] as well as their relationship with obesity risk [7-10]. Therefore, classifying supermarkets separately from grocery stores may be important to accurately assess how the food environment contributes to obesity.

Comparisons of detailed store attributes by classification match status in our study revealed two particularly important findings. First, correctly classified supermarkets, grocery stores, and convenience stores in the business lists had more cash registers (a proxy for store size), different types of service counters (supermarkets and grocery stores only), and a large selection of fresh fruit and vegetables (supermarkets and grocery stores only) compared to their misclassified counterparts, implying that larger supermarkets and grocery stores tended to be more accurately classified in both business lists. Second, misclassified supermarkets, grocery stores, and specialty food stores tended to be atypical, such as (for supermarkets and grocery stores) being attached to gas stations or carrying a relatively small number of fresh fruit and vegetables (for specialty food stores), or carrying a relatively large selection of fresh fruits and vegetables (likely produce markets) or fresh meat (likely meat markets).

### Limitations

We acknowledge the limitations of our study, which include that our ground-survey data is based on one metropolitan urban area in the United States at a single time point, and thus, whether the results of this study can be generalized across the United States and across time is not known. We also acknowledge that the standard classification of food stores based on store attributes is still debated in the literature [29], and thus, our classification may not be generally accepted. Despite these limitations, this study improves our understanding about two large commercial business databases with regard to the extent to which researchers may be able to rely on the classification of food outlets in such databases. Recently, researchers have highlighted the need to develop methodologies to address classification errors when using commercial data sources [29], and the importance of understanding the implications of these errors on research findings.

### Implications for future research

For future studies, our results imply that researchers can rely on the classification of D&B and InfoUSA when focusing on supermarkets or grocery stores. This is because both commercial lists performed moderate to well in correctly classifying supermarkets and grocery stores, and no systematic bias in terms of neighborhood characteristics was found in whether supermarkets and grocery stores were correctly classified. However, researchers should be aware that some atypical forms of food stores such as supermarkets and grocery stores attached to a gas station were likely to be misclassified in both lists. If those rather uncommon forms of food stores are more likely to be found in some neighborhoods, the classification of food stores in those secondary databases for such uncommon types of food stores may be less reliable. For example, in our sample, such atypical type of stores were more likely to be found in predominately Black tracts. Furthermore, given our finding that the racial and ethnic composition of the neighborhood was a statistically significant predictor for the classification bias for convenience stores and specialty food stores in both lists, research results focusing on convenience stores or specialty food stores are subject to some bias when they are derived using the classification in those commercial datasets.

## Conclusions

We built on the previous literature and assessed classification bias for food stores in two widely used commercial business lists in the United States. By using detailed outlet attributes to classify each food store based on actual observations inside the premises of retail outlets, we showed that potential classification bias in the business lists existed, particularly for some atypical forms of supermarkets, grocery stores, and specialty food stores. We also found that the classification bias systemically varied by the racial and ethnic composition of a census tract for convenience stores and specialty food stores whereas no systematic bias was found for supermarkets and grocery stores. Given the limited feasibility of collecting data on the food environment by ground survey on a large scale, it is important to understand the extent to which such secondary data are subject to classification error.

## Abbreviations

BMI, Body mass index; D&B, Dun & bradstreet; Chicago MSA, Chicago metropolitan statistical area; SIC, Standard industrial classification; NAICS, the North American Industry Classification System.

## Competing interests

The authors declare that there are no conflicts of interest.

## Authors’ contributions

EH, LMP, and SNZ contributed to study concept and design. EH analyzed data and EH and LPM interpreted data. EH drafted the manuscript. LMP and SNZ, LR, and PO-V and FJC provided critical review of the manuscript. FJC obtained funding for this study. EH and LMP were responsible for statistical analysis. All authors read and approved the final manuscript.
